# Supplementary material for: Inequity in Access and Delivery of Virtual Care Interventions: A Scoping Review
Source: Int J Environ Res Public Health. 2022 Aug 1;19(15):9411. doi: 10.3390/ijerph19159411 (PMC9367842; doi:10.3390/ijerph19159411)
Supplement: Supplementary file 1 [file ijerph-19-09411-s001.zip › ijerph-1755202-supplementary.pdf]

Supplementary Table S1: Preferred Reporting Items for Systematic reviews and Meta-Analyses extension for Scoping Reviews (PRISMA-ScR) Checklist

SECTION	ITEM	PRISMA-ScR CHECKLIST ITEM	REPORTED ON PAGE #
**TITLE**			
Title	1	Identify the report as a scoping review.	1
**ABSTRACT**			
Structured summary	2	Provide a structured summary that includes (as applicable): background, objectives, eligibility criteria, sources of evidence, charting methods, results, and conclusions that relate to the review questions and objectives.	1
**INTRODUCTION**			
Rationale	3	Describe the rationale for the review in the context of what is already known. Explain why the review questions/objectives lend themselves to a scoping review approach.	1-3
Objectives	4	Provide an explicit statement of the questions and objectives being addressed with reference to their key elements (e.g., population or participants, concepts, and context) or other relevant key elements used to conceptualize the review questions and/or objectives.	3
**METHODS**			
Protocol and registration	5	Indicate whether a review protocol exists; state if and where it can be accessed (e.g., a Web address); and if available, provide registration information, including the registration number.	3
Eligibility criteria	6	Specify characteristics of the sources of evidence used as eligibility criteria (e.g., years considered, language, and publication status), and provide a rationale.	3-4
Information sources*	7	Describe all information sources in the search (e.g., databases with dates of coverage and contact with authors to identify additional sources), as well as the date the most recent search was executed.	3-4
Search	8	Present the full electronic search strategy for at least 1 database, including any limits used, such that it could be repeated.	3-4
Selection of sources of evidence†	9	State the process for selecting sources of evidence (i.e., screening and eligibility) included in the scoping review.	4-5
Data charting process‡	10	Describe the methods of charting data from the included sources of evidence (e.g., calibrated forms or forms that have been tested by the team before their use, and whether data charting was done independently or in duplicate) and any processes for obtaining and confirming data from investigators.	5
Data items	11	List and define all variables for which data were sought and any assumptions and simplifications made.	5
Critical appraisal of individual sources of evidence§	12	If done, provide a rationale for conducting a critical appraisal of included sources of evidence; describe the methods used and how this information was used in any data synthesis (if appropriate).	Not applicable
Synthesis of results	13	Describe the methods of handling and summarizing the data that were charted.	5

Supplementary Table S2: Characteristics of included studies

Study	Title	Country	Study setting	Study design	Study population/subject/participants	Characteristics of intervention	virtual care modality	Types of inequity issues addressed/identified	Main findings	Summary conclusion	Relevance to virtual care interventions
Abel 2018 [61]	Dual Use of a Patient Portal and Clinical Video virtual care by Veterans with Mental Health Diagnoses: Retrospective, Cross-Sectional Analysis	USA	This study was carried out among veterans from a retrospective cohort study evaluating technology adoption in VHA (Veterans Health Administration) users	Retrospective cross-sectional analysis	The study population includes 2,171,325 veterans with one or more mental health diagnoses who were users of services from the Veterans Health Administration.	Two types of virtual care were offered and reviewed - the video virtual care service and the electronic portal and health record.	i)Clinical video virtual care. ii)Electronic patient portal and personal health record.	Mental health problems, age and veteran status.	African American and Latino patients, those with lower incomes and diagnosed with schizophrenia or schizoaffective disorder were less likely to engage in either virtual care modality. Women were more likely to engage in either virtual care modality compared to men. Patients who engaged in both virtual care modalities were younger, more likely to be white and less likely to be from low incomes. Rural patients were more likely to use clinical video virtual care services than urban patients.	This study pointed that there is socio-economic, gender based and racial disparities in access to virtual care intervention that need to be addressed to ensure equity.	Virtual care interventions may care for patients with mental health problems via clinical video virtual care appointments.

Alam 2019 [32]	Determinants of access to eHealth services in regional Australia	Australia	Western Downs Region in Southeast Queensland (~200km from a metropolitan city)	Cross-sectional questionnaire based household survey	A total of 390 randomly selected adults living in the area where the study is set.	N/A	eHealth services - use of internet and related technologies for healthcare services	Residence based inequity	Approximately 78% of the participants had access to virtual care services and access was lower among disadvantaged populations. Participants who reported being middle aged, living in a house with 3-4 people, and having broadband Internet access and good digital literacy were more likely to access virtual care services. Participants who reported lower levels of education, low socioeconomic status and living very remotely were less likely to access virtual care services.	This study reported on a variety of factors that increase and decrease access to virtual care services in populations living regionally.<br><br>A majority of the regional population sampled in this study had access to virtual care services.	Virtual care interventions may care for patients who live in regional areas, and thus it is important to understand what influences access and use of these services among regional populations.

Arighi 2021 [37]	Facing the digital divide into a dementia clinic during COVID-19 pandemic: caregiver age matters	Italy	Neurodegenerative Diseases Unit, Dementia Clinic, Alzheimer's Centre.	Cross-sectional analytic	Patients with cognitive impairment.<br><br>The sample size is 108.	The virtual care intervention in this study involved contacting patients via a video call to perform a telemedicine neurological evaluation. The intervention was assessing what contributed to a successful telemedicine interaction and to a failed telemedicine interaction.	Video calls to conduct telemedicine neurological evaluations using Microsoft Teams.	Access to technology, ability to use technology.	This study found that issues such as lack of devices (computers, phones or tablets) with internet connection and poor internet connections were the main causes of failed virtual care. virtual care interactions were more likely to be successful if the patient was in the presence of a younger caregiver for the interaction. Factors such as age, gender or education level were not associated with success or failure of the virtual care interaction.	The study findings suggests that virtual care services could be made more equitable if patients can be in the presence of a younger person for their virtual care appointments. It also suggests that access to devices and internet connection are essential for accessing virtual care services.	This study suggests that it may be useful to encourage the patients to have a younger caregiver or family member present with them for their virtual care appointments. Patients should also have good internet connection.

Arora 2013 [35]	Dismantling sociocultural barriers to eye care with tele-ophthalmology: lessons from an Alberta Cree community	Canada	A community-based health clinic for Aboriginal Canadians in Alberta.	The study type is not clear.	Aboriginal Canadian patients who attended the community-based health clinic.<br><br>The paper does not provide the study sample size.	The intervention refers as 'tele-ophthalmology' - the remote provision of eye care. It involves a remote diabetic retinopathy screening program delivered to patients at a community-based health clinic.	Remote screening for diabetic retinopathy.	Cultural barriers, access to health services, and remote geographical locations.	The study found that establishing the community-based clinic as a setting for virtual care services improved the patients' access to tele-ophthalmology.	This study showed that virtual care services for patients who are Indigenous or living remotely can be made more equitable if these services can be accessed in a culturally appropriate community-based healthcare clinic.	This study may not be particularly relevant to virtual care intervention. This is because while the intervention is a virtual care modality, it was delivered in a healthcare setting in a community-based clinic. virtual care interventions focus seems to be caring for patients in at home settings.

Blundell 2020 [56]	Disparities in telemedicine access for Spanish-speaking patients during the COVID-19 crisis	USA	An urban academic outpatient paediatric dermatology clinic.	Retrospective analysis of clinic data.	Patients of the paediatric dermatology clinic whose appointments scheduled in March-July 2019 and/or March-July 2020. The sample size was 53 Spanish speaking patients and 1025 non-Spanish speaking patients.	Telemedicine appointments in place of face-to-face outpatient appointments at the paediatric dermatology clinic.	The appointments were scheduled electronically, communications took place via an electronic patient portal, and appointments took place over telemedicine (whether this is a phone call, or a video call is not specified in the paper).	CALD barriers, particularly non-English speaking and lower income level.	The study found that having a valid email address on record for Spanish speaking patients was an important factor in delivering virtual care services. It was reported that Spanish speaking patients were less likely to have a valid email address on their record compared to non-Spanish speaking patients. This study also reported that Spanish speaking patients found communication via text messaging easier compared to communication via an electronic patient portal.	This study reported its main finding to be the importance of being aware of patient's communication preferences, particularly for patients whose primary language is not English. The study also suggested that patients being connected via email was an important factor in determining virtual care access.	This study could help inform virtual care interventions' procedures for communicating with patients whose primary language is not English.

Campos-Castillo 2021 [42]	Racial and ethnic differences in self-reported virtual care use during the COVID-19 pandemic: a secondary analysis of a US survey of internet users from late March	USA	Survey of Internet users.	Secondary analysis of a cross-sectional nationally representative survey of internet users.	The study population was a nationally representative sample of 10624 adults living in US households.	This study surveyed whether participants accessed virtual care (via synchronous and asynchronous methods) during the COVID-19 pandemic.	Synchronous (telephone and video calls) and asynchronous (messaging, emails or text messaging).	Race and ethnicity based inequity	This study firstly reports that in March 2020, black participants were more likely to use virtual care services compared to white participants. This was particularly found in black participants who reported being fearful of the COVID-19 pandemic.<br><br>This study notes that having a range of virtual care modalities available (synchronous and asynchronous) is important to ensure equitable access for all users.	This study report that black people were more likely to use virtual care services compared to white people durng the COVID-19. The study also highlights that different virtual care modalities are essential to to ensure equitable access for all users.	Evidence from this study is not particularly relevant to virtual care interventions. However, this study highlights the importance to increase access to virtual care services for minority ethnic groups.

Chunara 2021 [62]	Telemedicine and healthcare disparities: a cohort study in a large healthcare system in New York City during COVID-19	USA	New York University Langone Health Service	Cohort study	Patients from New York University Langone Health Service between 19/03/2020 and 30/04/2020. The sample size was 140184.	This study describes the disparities in people who accessed healthcare via telemedicine during the COVID-19 pandemic.	Telephone and video call for urgent and non-urgent ambulatory care.	Age, gender, race, ethnicity, non-English speaking, location, education, income and comorbidities.	This study showed that the number of black patients who accessed virtual care services was increased. Yet, black patients were less likely to access virtual care services compared to white patients. The study identified that younger, female black patients drove this increase.<br><br>The study showed that black people who did access virtual care services were sicker compared to white people who accessed virtual care services.<br><br>This study showed that English speaking patients were much more likely to access virtual care services compared to non-English speaking patients.	This study provides evidence that white patients are more likely to access virtual care services than black patients. This study also shows that English speaking patients are more likely to access virtual care services than non-English speaking patients.	This study demonstrates the need to carefully tailor services to people from minority backgrounds in order to ensure they can access virtual care services at the same level that white English speaking patients can.

Darrat 2021 [63]	Socioeconomic Disparities in Patient Use of virtual care During the Coronavirus Disease 2019 Surge	USA	Department of Otolaryngology-Head & Neck Surgery, Henry Ford Hospital. Detroit, Michigan.	Cohort study	All patients who had encounters (telephone and virtual), with the Department of Otolaryngology-Head & Neck Surgery at Henry Ford Hospital during 17/03/2020 - 01/05/2020. The sample size was 1162.	The virtual care intervention in this study incorporated virtual visits and telephone visits.	Virtual (internet-based video calls) appointments and telephone appointments.	Insurance status, socioeconomic status, age, sex and marital status.	This study found that older patients, black patients, patients with no insurance coverage, unmarried and patients from low socioeconomic backgrounds were less likely to access virtual appointments.<br><br>Patients who were older, with no insurance coverage and who were from low socioeconomic backgrounds were more likely to engage via telephone appointments rather than virtual appointments. Telephone appointments are an acceptable virtual care modality, although this study reports that virtual appointments provide a better standard of care.	The study suggests that virtual care initiatives need to include patient education and training to foster access and acceptance of these services. The study suggests that this should be targeted to older patients. The study also notes the importance of considering internet access among target populations when developing virtual care services.	This study has provided important information regarding the use of virtual appointments compared to telephone appointments and points out that some demographic groups are more likely to use telephone appointments. This could be useful for informing virtual care interventions' planning of services.

Eberly 2020 [64]	Patient Characteristics Associated With Telemedicine Access for Primary and Specialty Ambulatory Care During the COVID-19 Pandemic	USA	Department of Medicine, Hospital of the University of Pennsylvania. Virtual care was offered in: cardiology, pulmonology, hemato-oncology, primary care, rheumatology, gastroenterology, infectious diseases and nephrology.	Cohort study	80780 patients who completed telemedicine visits in the study period (16/03/2020-11/05/2020). The patients live across large urban, suburban and semirural areas in Pennsylvania and New Jersey.	The telemedicine intervention in this study incorporated video calls and telephone calls. Patients received reminder calls and instructions for setting up video technology for their visit.	Video call appointments and telephone appointments.	Age, sex, race, ethnicity, preferred language, insurance, income, comorbidity status.	This study found that patients who completed telemedicine visits (either via video call or telephone appointments) were more likely to be female, have insurance, be English speaking and come from non-Asian backgrounds. Black, Latina and lower socioeconomic patients were less likely to engage in video call appointments. Patients who were Asian, older and whose primary language was not English were less likely to engage with either type of telemedicine visit (video call or telephone call). Importantly, this study notes that there does not seem to be better patient care outcomes from video appointments compared to telephone appointments. However, clinicians prefered video appointments.	This study identified inequality in virtual care services including video care and telephone appointments in a hospital setting based on age, sex, race ethnicity, language spoken and socioeconomic status. rather than telephone appointments. These included being younger, white and having a higher socioeconomic status.	This study points out that some demographic groups are more likely to use telephone appointments, where others are more likely to use video call appointments. This could be useful for informing virtual care interventions' planning of services.

Ernsting 2019 [39]	Associations of Health App Use and Perceived Effectiveness in People With Cardiovascular Diseases and Diabetes: Population-Based Survey	Germany	Survey of Internet users.	Cross-sectional survey.	German people aged 35 and over, with cardiovascular disease and or diabetes.<br><br>The sample size is 1500.	The telemedicine intervention in this study is a mobile health app/eHealth app.	Mobile health app/eHealth app.	Sociodemographic, health behaviours, health literacy and eHealth literacy, and presence of cardiovascular disease and or diabetes.	The study found that people who engaged with mobile health apps/eHealth apps were more likely to be younger, female, have higher levels of education, and were more likely to engage in health behaviours such as physical activity. The study found that app users have higher levels of eHealth literacy compared to non-app users. In addition to this, users who had higher levels of eHealth literacy more often believed the apps were effective.	An important finding of this study is that eHealth literacy is essential for use of mobile health apps, and this should be considered in health education strategies and in planning virtual care initiatives.	The information regarding equity in accessing m-health/e-health apps is relevant for virtual care in general and can be used to inform virtual care interventions' virtual care services.
Ferguson 2020 [72]	Virtual Care Expansion in the Veterans Health Administration During	USA	Veterans Health Administration	The study type is not clear.	Veterans who had outpatient encounters during the early phase of the COVID-19	The telemedicine intervention in this study incorporates virtual care including phone and video	Virtual appointments - phone and video appointments.	Sociodemographic and clinical needs.	This study found that patients who had higher disability levels and more chronic conditions were more likely to receive virtual care in the pandemic. It was also reported that older patients were less likely to use video appointments	This study showed that in the early phase of the COVID-19 pandemic, veterans who had higher clinical and social needs	This study was particularly focused on the early phase of the COVID-19 pandemic and how

	the COVID-19 Pandemic: Clinical Services and Patient Characteristics Associated with Utilization				pandemic.<br><br>The sample size is	appointments.			compared to younger patients. Furthermore, rural and homeless veterans were also less likely to use video appointments compared to veterans who lived in urbans areas and were not homeless.	were more likely to use virtual health services. Another important finding was that older veterans and veterans who lived rurally and were homeless, were less likely to access video appointments.	veterans engaged with virtual care services at that time. However, the findings from this study are relevant for virtual care interventions to apply when planning their services for older patients.

Foley 2020 [33]	Exploring access to, use of and benefits from population-oriented digital health services in Australia	Australia	Participants were recruited from the online health information website 'Healthdirect Australia', and waiting rooms of 'General Practice (GP)' services	Mixed methods including Quantitative surveys and qualitative telephone interviews	441 participants were included from the online health information website 'Healthdirect Australia' and 40 qualitative telephone interviews were conducted among participants recruited from the waiting rooms of 'General Practice (GP)' services.	N/A	eHealth Literacy, digital health services	Equity issues related to sociodemographic characteristics and trust.	The study found that being older, having low socioeconomic status, being male, being Aboriginal or Torres Strait Islander and having no tertiary education was negatively associated with access to digital health services. The study also pointed that trust in digital health services has an important influence on their use.	The study summarizes that individual without tertiary education, identifying as Aboriginal or Torres Strait Islander, or from socioeconomically disadvantaged areas were less likely to access digital health services.	It is very important form virtual care interventions' perspective that these issues are urgently addressed to prevent exacerbating already existing health inequities in Australia.

Gilson 2020 [57]	Growth of Ambulatory Virtual Visits and Differential Use by Patient Sociodemographic at One Urban Academic Medical Center During the COVID-19 Pandemic: Retrospective Analysis	USA	University of Chicago Medical Centre.	Retrospective analysis of clinic data.	Patients who had clinic encounters between 15/03/2020 and 31/05/2020.	The virtual care intervention in this study incorporated video visits and telephone visits.	Virtual (including video) appointments and telephone appointments.	Race, sex, age and insurance coverage.	This study found that younger patients (aged 0-17) were less likely to attend a virtual appointment. Men were less likely to attend a virtual appointment than women. There was no difference between black and white patients in terms of engaging with virtual appointments. Patients who categorized their race as 'other' were more likely to attend a virtual visit compared to white patients. Patients with insurance were more likely to attend a virtual appointment compared to patients without insurance.	There are a variety of factors including age, sex, race, and insurance coverage that were shown to impact engagement with virtual appointments in this study.	This study has provided important and useful information regarding the use of virtual appointments and points out that some demographic groups are more likely to engage with virtual appointments than other. This could be useful for informing virtual care interventions' planning of services.

Gordon 2018 [43]	Older adults' readiness to engage with eHealth patient education and self-care resources: a cross-sectional survey	USA	Kaiser Permanente Medical Care Program in Northern California.	Cross-sectional survey.	Members of Kaiser Permanente Medical Care who are aged 65-79 years with English as their primary language. There were white, black, Hispanic, Filipino and Chinese participants. The sample size is 5420.	The virtual care intervention in this study incorporated web-based and other digital technologies to obtain health information and advice.	Web-based and other digital technologies for online health information, and mobile health monitoring tools.	Inequity based on age, sociodemographic, sex, race, ethnicity, level of education, self-rating of health and use of medication and use of digital technology on the internet.	About three-fourth of the sample could easily access a device with an internet connection. However, ease of access declined with age and was reported more often in white participants.<br><br>Nearly all participants reported being able to access the internet at home, and a majority reported being able to access the internet by themselves or with help from someone to research health information on the internet. The participants who responded in this way could also use email for communication with ease. Those aged 65-69 years were more likely to be internet users, as were white and Chinese participants.	Overall, this study shows that a majority of this sample of older aged patients can access the internet easily. This is an important consideration for virtual care services.	This study provides virtual care interventions with some data regarding how easily older patients from different ethnic backgrounds can access the internet, which is an important consideration for virtual care services.

Gordon 2016 [71]	Differences in Access to and Preferences for Using Patient Portals and Other eHealth Technologies Based on Race, Ethnicity, and Age: A Database and Survey Study of Seniors in a Large Health Plan		Kaiser Permanente Medical Care Program in Northern California.	The study type is not clear. It seems to be both a cohort study (analysing a database) and a cross-sectional study (conducting a survey).	Two studies were conducted. Both used this population: Members of Kaiser Permanente Medical Care who are aged 65-79 years with English as their primary language. There were white, black, Hispanic, Filipino and Chinese participants.	The virtual care intervention in this study is based on eHealth technologies such as patient portals.	eHealth technologies such as a patient portal - used to interact with health care system.	Race, ethnicity, and age.	Older participants were significantly less likely to be registered to use the online patient portal. Older participants who were registered to use the online patient portal engaged with this service much less than younger participants who were registered.<br><br>White and Chinese articipants were the most likely to use and engage with the online portal. Black, Latino and Filipino participants, as well as participants aged over 75, were significantly less likely to own digital devices, be able to use the internet and email communication, and be willing to use the online portal.<br><br>Overall, even among participants who could use the Internet, most preferred	Older patients and ethnically diverse patients are less likely to use the online portal. Ethnically diverse patients are less likely to be able to access digital health services. Most older patients preferred non-digital modalities.	This study provides virtual care interventions with some data regarding how older patients from different ethnic backgrounds feel about accessing digital health services, which is an important consideration for virtual care services. It also provides virtual care interventions with data regarding

					The first study was a database analysis, and the sample size was 213173.<br><br>The second study was a survey of participant s from the above sample. The sample size was 2602.				non-digital interactions with their health care system, i.e., not use the online portal.		how easy it is for older patients from different ethnic backgrounds to access digital health services.

Guendelman 2017 [69]	Listening to Communities: Mixed-Method Study of the Engagement of Disadvantaged Mothers and Pregnant Women With Digital Health Technologies	USA	Low income communities in San Francisco, New York and Kentucky.	Mixed methods. 14 focus groups conducted, in total 92 participants. Quantitative data collected at FG	92 first-time pregnant women and mothers of children under the age of 5. Recruited from community clinics, federally qualified health centres, Women, Infants and Children (WIC) clinics and NGOs. Age 25-32years, The majority was black	Descriptive study, with statistical modelling	Websites, apps, wearables, social networks, video chats and patient portals	Socio-economic status, A particular vulnerable group (pregnant and mothers). Functional health literacy opposed to eHealth literacy	Among the low-income study population, the study found a very high access to smartphones and computers. Number of internet health-seeking behaviour in the past 12 months: 97% of participants did internet search for health information in the past 12 months. Of them, 25% conducted a high number of searches (>9). But a low use of internet or other digital tools for health management practices (accessing personal data, making appointments, email communication, video chats etc.); 27% used 4 or more practices while 42% engaged in none.<br>49% of non/low users are potential users with interest in using digital health management tools, but some reported not being taught how to do so.<br>eHealth literacy increase	Web-based information search was widespread, while the use of digital health management practices was far less common in the study group. A significant relationship between health search activities and digital-heath management practices. The study demonstrated eHealth literacy is strongly associated with internet search activities, internal orientation (motivation to	Pregnant women and mothers demonstrated a widespread behaviour of internet health information seeking. But to go beyond search activities and actively engage with digital health management practices, they need encouragement from health professionals to endorse such behaviour with credible

					or Hispanic, married or have partner, college education, unemployed or not in labour force, on Medicaid. Residing in the study area and able to read, write and speak English.				associated with 3% increase in number of searches (beta=.03, 95% CI 0.00-0.06). Internal health orientation scores corelated with the total no. of digital health management activities (beta=.13, 95% CI 0.02-0.24). Trust in digital information was associated with the current level of use (p=.05) .	engage in healthy attitudes, beliefs and behaviour) was more strongly associated with digital health management practices. Trust in digital information is associated with potential adoption of digital tools. Relative contributions of these factors to be explored to develop better tools and intervention.	information sources and give practical tips on how to use digital health management tools.

Hansen 2019 [40]	Inequalities in the Use of eHealth Between Socioeconomic Groups Among Patients With Type 1 and Type 2 Diabetes: Cross-Sectional Study	Norway	Monitoring and follow-up of diabetes patients	Cross sectional	Members of Norwegian Diabetes Association (18-89 years) with Type 1 and Type 2 DM. 1,063 participants in total	N/A	• Apps,<br>• Search engines,<br>• Video services,<br>• Social media sites	Socioeconomic status - education, household income, age, gender, marital status.	87% of T1D and 78% of T2D (82 % in total) used 1 or more form of eHealth in the previous year. Search engines is commonest (78%), followed by Apps (53%), social media (38%) and video services (18%). Strong association of higher level of education and use of search engine (OR 3-6 compared to low education group). This may be explained by the capabilities of the group to seeking out, finding, understanding and utilising the information. No association of education level with the use of Apps, social media or video services. Higher income associated with the use of Apps in both groups (OR 3), may be reflecting the cost to download. Among T1D group, an association between lower income and video service use.	Information though Apps, social media and video services may be better choice when targeting the lower educational group. Be aware of inequalities in eHealth use to design communication strategies to different target groups, particularly that of education level.	Communication strategies to varying educational level to be developed, eg; tailor the information or select platforms to the expected audience.

Jaffe 2020 [65]	Health Inequalities in the Use of virtual care in the United States in the Lens of COVID-19	USA	Primary care during Pre Covid and at the beginning of the pandemic (March 2019 and March 2020)	Retrospective cohort study. Used linked data from National Health and Wellness Survey (NHWS) and Komodo Health's encounter data.	35,376 persons aged >18 years, who had more than >1 health care encounter in the study period.	N/A	Not clearly explained	Age, gender, ethnicity, marital status, employment status, geographic location (socioeconomic), urban/rural, Self-reported medical diagnosis (SRMD)	Virtual care clams increased 0.2% in March 2019 to 1.9% (845% increase) in March 2020 across the age groups. Younger people (18-44), urban people, female, Hispanic, married or living with a partner and employed had a higher usage of virtual care (p=0.005). People with cardiovascular or metabolic conditions associated with lower usage (51% virtual care encounter TE to 43% in-person encounter PE, p<0.001) and those with anxiety and depression used more (50% TE to 32% PE). After adjusting, age, marital status, geographic location and urbanisation, SRMD anxiety and depression were predictor for the use of virtual care.	virtual care could help reduce inequalities in health care access. However, inequalities were observed. Greater outreach, education, and infrastructure support are needed for older individuals, those residing in South (higher poverty) and those residing in rural areas.	Covid pandemic increased the use of virtual care. Use of virtual care seems to be age, marital status, socio-economic status. May need to see actual user patterns to optimise virtual care interventions hospital access.

Jiang 2020 [58]	Equal Access to Telemedicine during COVID-19 Pandemic: A Pediatric Otolaryngology Perspective	USA	Study conducted during the hospital clinic was closed for unurgent visits, commence on 19 March and lifted on 4 May 2020. Paediatric tertiary care clinic, attended by referral. Serves a urban metropolitan area with 3 million population	Retrospective case series. 6 week between 23 March and 1 May 2020. (OPD closed during this period)	1,495 patient data who accessed telemedicine and 1,983 patient data who had in-person visits of the period in 2019	Telemedicine services introduced 3 years ago was ramped up to address increase in its use after the pandemic started.	Video conference (Zoom integrating access to patients: electric medical record (EMR), which allows simultaneous recording to EMR.) Patient can access through the portal via PC or smart phone. Telephone only access was also available.	Age, gender, language, zip code of residence (poverty level by US census 2019), primary insurance plan.	None of variable in relation to telemedicine use demonstrated statistically significant differences. Spanish speaking patients had a higher rescheduling need compared to overall patients rescheduled, (17% to 12%, p=0,083). Authors explain tertiary paediatric care nature may contribute to these findings- parents tend to be younger and familiar with technology.	Language barriers exist. Language service, increased staff support and longer appointment time, video digital education. It is essential to evaluate, understand and address potential barriers to technology-based platforms for delivering care to prevent further disparities in access to healthcare.	Examine the access to virtual care by CALD population.

Kemp 2020 [59]	Barriers associated with failed completion of an acute care general surgery virtual care clinic visit	USA	Surgery dept's e-clinic (University of Michigan)	Patient electronic health records review. Examined the characteristics of patients who completed or not completed e-clinic visits.	199 post-operative follow-up encounters of emergency, urgent and elective patients managed by an acute care surgery and had been referred to E-clinic between Jan 2019 to June 2019.	N/A	Video or telephone visits. Use of electronic health record's secure virtual platform (Epic)	Inequity based on soicoeconomic status, medical characteristics, operation, readmission, reoperation/procedure, visit prior to scheduled e-clinic visit, mortality within 30 days of discharge	E visit completion rate was 78% (156/199). Video e-clinic 71%, telephone 29%. A higher proportion of Caucasian (87% to 74% p=0.04) and married patients completed e-clinic visits (49% to 26%, p<0.05). Cancellation or no-show associated by travel distance (Patients living 30 to 44.9Km away had a higher cancellation p<0.05). Higher frequencies of 30-day reoperations/procedure in the incomplete group(p<0.05).	E-clinic is safe and feasible method for providing postoperative care in well-selected patients. Racial disparities still exist in accessing virtual care. Patients' preference and comfort to be taken into consideration and suggest the importance of discussing with patients about how and who conducts E-clinic and (data) security.	Selection of patients to virtual care taking their preference, technical environment, support at home and their medical conditions into consideration is important for reducing the cancellation of virtual care clinic appointments.

Khoong 2020 [44]	Patient interest in and barriers to telemedicine video visits in a multilingual urban safety-net system	USA	Women's health and general medicine clinic in an urban safety-net system, where most patients are uninsured or Medicare insured. (Low socio-economic status, high racial/ethnic diverse population.)	Cross sectional phone survey and data review of patients' records.	298 called and 202 were reached. 88% identified as persons of colour and /or preferred on-English language (56%)- mainly Spanish. The survey was conducted in the patients' preferred language.	Patients who were interested in the video-visit was asked to download a video-visit application and tested completion of video-visits.	Video-visits (testing only)	Digital device with video capability, access to mobile data/internet/ and prior of smartphone applications. Age, language.	65% of the participants were interested and 54% completed a simulated video visit. People with <55 years completed video visits more than those older. Non-English speakers were more likely to be interested in video visits (76% to 51%, p<0.05). Of those who had incomplete video-visits, >30% reported data/internet access (45%), hesitancy about technology (36%), no access to device (33%) or belief that video visits were not better than telephone visits (33%). Younger people were more likely to have devices but reported video-visits were no better than phone visits. 25% of people who completed video visits needed support, particularly non-English/Spanish speakers (P<0.002) and older people (P <0.001).	Diverse low-income patients are interested in video visits, and many are able to complete simulated video visits. However, policies and infrastructure development are needed to address gaps in access to broadband or mobile data. Health care system/providers to provide technical assistance to older patients and to those with limited digital literacy.	Most people are interested in virtual care provided access to digital device and support are provided. Older patients and those with limited digital literacy need targeted support for equitable access to virtual care.

Leng 2016 [41]	The use of video consulting in general practice: semi-structured interviews examining acceptability to patients	Scotland	This study was carried out in three purposively chosen general practice in Lothian, Scotland	Cross-sectional	A total of 270 adult patients aged 18 years or above from three general practice of Lothian, Scotland were interviewed using a semi-structured questionnaire.	N/A	Patients perceptions with video consulting was assessed	Equity issue related to the sociodemographic characteristics of the participants were analysed	The study revealed that the patients under 60 years were over two times more likely to use video consulting (Odds Ratio (OR) 2.2, 95% CI 2.1–6.6). A positive trend was also observed between increasing computer proficiency and willingness to video consulting ($\chi 2$ = 43.97, $p < 0.0005$, n = 270). Also, patients who had used commercial video services (such as Skype) were approximately six times more likely to favour video consulting than those who had not (OR 5.9, 95% CI 3.5–9.9).	The study summarizes that despite possibilities of video consultation in primary care, its use can be compromised among older adults and less technically able person	It is important to consider the digital literacy level of the people and age of the participants while developing a virtual care intervention. Tailored approach can be useful can be of value in ensuring equity in this regard.

Li 2020 [38]	Patients' Perceptions of Barriers and Facilitators to the Adoption of E-Hospitals: Cross-Sectional Study in Western China	China	1 tertial hospital, 1 secondary hospital and primary health care centres (PHC) in Chengdu, the capital of Sichuan province in west China from June to August 2019.	Cross sectional, self - administered questionnaires or administered by a research investigator.	1108 patients attending the survey sites, aged 18 years and above. Of them, 1032 patients completed the survey in full. 76 incomplete surveys were excluded. No particular inclusion or exclusion criteria set.	N/A	e-hospitals that are administered by physical hospitals, where patients able to reach physicians in tertial hospitals via internet and are referred to PHCs or secondary hospitals.	Sociodemographic, medical, current, and past use of web-based health care service.	Facilitators: Convenience (95%) and access to skilled medical experts (72%) were main facilitators. Younger people (18-34 years) reported better health outcomes, protect privacy and self-management as facilitators compared to older people. Barriers: Overall, inability to operate technological device (67%), familiarity with face-to-face health care (30%) and doubts regarding authenticity and reliability of e-hospitals (10%). Inability to operate devices among older people >65 years (87%), concerns over authenticity and reliability (35%) among people aged 35-49 years, and accustomed to face-to-face consultation (75%) and concern about insurance reimbursement (25%).	Efforts to increase the adoption of e-hospitals should focus on making target populations accustomed to web-based health care services while maximising ease of use of providing assistance for technological inquiries.	Tailored App design with technical services to increase usage among elderly population. The uptake and continued use of virtual hospital will depend on the perceived quality of care patients receive, as this study indicate that experience is a predictor for the uptake of virtual care services.

Mangin 2019 [36]	Multimorbidity, eHealth and implications for equity: a cross-sectional survey of patient perspectives on eHealth	Canada	Primary care clinics under the McMaster University sentinel and information collaboration (MUSIC) network. Mid-Dec 2014 to Mid Jan 2015.	Cross sectional survey collecting quantitative and qualitative data.	Consecutive patients attending primary care appointments with physicians who are part of MUSIC primary care practice-based research network. In total, 693 patients completed the survey.	N/A	eHealth defined as "activity in booking appointments, gathering health information, communicating with your family health team and personalised monitoring and information around your health online".	Demographic, multimorbidity, home internet access, degree of confidence using internet, types of devise used, level of interest in eHealth and concerns about eHealth and privacy issues.	87% had internet access at home, which decreased significantly with age (p<0.001). 58% of participants expressed interest in eHealth and 20% expressed disinterest. People on 5 or more medications and those over 70 years were less likely to be interested in eHealth. 73% of participants had concern over privacy, which was the same across all the age groups. People on 5 or more medications were less likely to be concerned with privacy. Qualitative data indicated concern over data security/privacy and loss of human connection/interaction and communication with clinicians as 2 main concerns.	Multimorbidity is strongly associated with less interest, less access and less comfort in using eHealth. Privacy issues need to be explained well. Concern over loss of connection/interaction with clinicians is an important issue to address, particularly with people with multimorbidity, in primary care context where patient-centred care is essential.	Multimorbidity may be an issue for population covered by SLHD too. eHealth strategies addressing people with chronic diseases need to take the burden of treatment that patients have into consideration to make it more accessible and effective.

Marrie 2019 [5]	Use of eHealth and mHealth technology by persons with multiple sclerosis	USA	North American Research Committee on MS Registry (NARCOMS)	Cross sectional based on the survey data.	Patients enrolled in the NARCOMS spring survey, 2017. 7281 responded to the survey. (all p<0.001).	N/A	eHealth, mobile Apps, virtual care	Sociodemographic and lifestyle characteristics, use of eHealth technologies, connection and devices, software/ Apps, benefits of using Apps. Communication with health care providers electronically.	91% of all respondents (6423) had reported any internet use. Overall, 84% (5,408) exchanged medical information with a health professional, often using secure online portal (27%), followed by email (21%). The interest in exchanging health information electronically with a provider varied; test results being highest (70%) followed by appointment reminders and diagnostic information. Of the 5,529 smartphone and tablet users, 46% used a mHealth App. Increase smartphone use associated with higher income, being single and any physical activity, 3 or more comorbidity while advanced age, smoking and disability were associated reduced use of smartphone.	Use of mHealth apps is perceived to have health benefits. However, use of eHealth and mHealth technologies varies substantially with sociodemographic factors, and health care providers need to be aware of these disparities as these technologies are increasingly leveraged in health care setting.	Be aware of disparities in the use of virtual care.

Nelson 2016 [45]	Disparities in the use of a mHealth medication adherence promotion intervention for low-income adults with type 2 diabetes	USA	Participants were recruited from a Federally Qualified Health Center (FQHC) in Nashville, Tennessee.	Cross-sectional	A total of 80 adult patients were recruited who were at least 18 years of age, English-speaking, diagnosed with T2DM, prescribed diabetes medication (s), owned a cell phone with SMS capability, and had a Social Security number	The MED (Messaging for Diabetes) intervention was designed to improve medication adherence for T2DM. It has 3 components: 1) a unique, daily, one-way text message; 2) a daily, two-way text message, and 3) an automated weekly IVR call.	Text messaging and automated voice call	Equity issues related to age, gender, race, income, and health literacy was addressed	The probability of responding to texts tended to increase from about age 25 until roughly age 50 years, and then appeared to decrease as age increased. Compared to White participants, non-White participants had a 63% decreased relative odds (AOR: 0.37, 95% CI, 0.19-0.73) of participating in voice calls. In addition, lower health literacy was associated with a decreased odds of participating in voice calls (AOR=0.67, 95% CI, 0.46-0.99, P=.04).	Racial/ethnic minorities, older adults, and persons with lower health literacy appeared to be the least engaged in a mHealth intervention.	To have more equitable services, virtual care interventions should focus on to provide more focus on to aged participants. They also need to make strategies to involve racial minority population as well as improving virtual care literacy among the participants.

Pierce 2020 [46]	Disparities in use of virtual care at the onset of the COVID-19 public health emergency	USA	Academic medical Centre, University of Missouri Health System, MU Health	Cross-sectional	The study includes all finalized ambulatory clinic encounter documentation completed Between 17 March to 16 April 2020. Final analysis included 7742 encounters representing 3938 face-to-face encounters and 3804 virtual care encounters.	Encounters with charges for services associated with a virtual care appointment type was classified as virtual care. Of the virtual care encounters, 2937 were full audio-video and 867 were audio-only.	Both audio-video and audio-only consultations	Equity analysis was based on region, sex, race and payment type.	virtual care visit was higher among aged 65 years and above (OR 1.21, 95% CI 1.05–1.40), female (OR 1.15, 95% CI 1.04–1.26) and those who were not under private insurance. virtual care visits were less frequent among those who were residing in rural areas (0.81, 95% CI 0.74–0.90) and who were Black (OR 0.65, 95% CI 0.56–0.75) or of another race (0.64, 95% CI 0.50–0.82) compared to those of white.	This paper pointed that, disparities are existed in virtual care use in terms of age, race, residence and payer at the onset of the COVID-19 in USA. It suggested for future research to clarify underlying causes these disparities and to inform policymaking during the COVID-19 emergency and beyond.	This study has identified diverse race and rural residence as a significant factor for seeking less virtual care. virtual care interventions also need to make strategy to engage patients of Culturally and Linguistically Diverse (CALD) Background and those who were residing in remote areas.

Potdar 2020 [47]	Access to internet, smartphone usage, and acceptability of mobile health technology among cancer patients	USA	The study was conducted in the outpatient clinic and infusion center at an academic medical center in Philadelphia	Cross-sectional	A total of 151 cancer patients attending the outpatient centre and infusion centre were included in the study	N/A	N/A	Equity issues in terms of education and age was considered	Participants aged 61–70 years (OR 0.24, 95%CI 0.07–0.90), 71–80 years (OR 0.05, 95%CI 0.01–0.23), and > 80 years (OR 0.04, 95%CI 0.01–0.22) were significantly less likely to utilize a daily mHealth application than individuals < 50 years. Also, acceptability of a daily mHealth application was significantly higher in patients with a college-level degree (OR 2.78, CI95% 1.25–5.88).	The acceptability of daily mHealth application was significantly higher among relatively educated and younger patients.	While looking to address the equity issues, virtual care interventions also need to consider that the use of this service would be lower among relatively aged and less educated people.

Rodriguez 2021 [48]	Differences in the use of telephone and video telemedicine visits during the COVID-19 pandemic	USA	Mass General Brigham (MGB) in Boston, Massachusetts, a large, integrated health system with 16 member organizations across New England	Cross-sectional	231,596 visits to primary care and specialty practices of 162,102 adult patients aged 18 years or above that includes telephone, video, and in-person visits between March 1 and June 1 2020 were analysed.	The study analysed health service visits in a large, integrated health system that includes telephone, video, and in-person visits.	Both telephone and video visits were considered	Age, race, internet access, education, socioeconomic status was considered while assessing the equity in accessing virtual care intervention	Patients who were older than 65 years (adjusted odds ratio [AOR], 0.41; 95% CI, 0.40-0.43), Black (AOR, 0.60; 95% CI, 0.57-0.63), Hispanic (AOR, 0.76; 95% CI, 0.73-0.80), Spanish-speaking (AOR, 0.57; 95% CI, 0.52-0.61), living in areas with the lowest broadband internet access (AOR, 0.93; 95% CI, 0.88-0.98), lowest median income (AOR, 0.49; 95% CI, 0.46-0.52), and lowest educational attainment (AOR, 0.84; 95% CI, 0.80-0.88) were less likely to use video visits.	Older age, non-white background, lack of access to internet, less educational attainment and poor socioeconomic status were significant factors in inadequate accessing to the video visits.	Efforts are needed to ensure access to virtual care intervention among the CALD population and those who are aged, and with poor e-health literacy. It is also important that people with low socioeconomic status and lesser access to internet are supported to have access to the services.

Schifeling 2020 [60]	Disparities in Video and Telephone Visits Among Older Adults During the COVID-19 Pandemic: Cross-Sectional Analysis	USA	Two primary care clinics in Colorado	Retrospective cross-sectional study	Electronic health record (EHR) review of 190 visit of patients aged 75 years or over	Information of 190 appointments were collected from EHR chart review, that includes 100 telephone visits and 90 video visits.	Both telephone and video visits were considered	Equity issues in terms in terms of age, race, presence of caregivers, requirement of interpreters and receipt of Medicaid was considered	Patients who had video visits were younger (mean 81.3 years, SD 6.4 years vs mean 83.5 years SD 5.9 years; P=.01), and more likely to have a caregiver present during the visit (n=31, 64.6% vs n=17, 35.4%; P=.01) compared to patients who had telephone visits. Non-White patients, patients who needed an interpreter, and Medicaid beneficiaries, were less likely to have video visits than White patients, patients who did not need an interpreter, and non-Medicaid beneficiaries (P=.003, P=.01, P<.001, respectively).	Relatively older people, non-while background and those who need an interpreter are less likely to use videos visits. Presence of caregiver plays a positive role in increasing video visits.	In order to reduce inequity of access to virtual care services, particularly among the older adults, presence of educated caregiver can play significant positive role. It is also crucial that strategies are being developed to engage more people from CALD background in virtual care services.

Severe 2020 [49]	Factors Influencing Patients' Initial Decisions Regarding Telepsychiatry Participation During the COVID-19 Pandemic: Telephone-Based Survey	USA	The study was carried out in the Outpatient Psychiatry Clinics at the University of Michigan health care system, known as Michigan Medicine	Cross-sectional	The study participants included a total of 244 patients whose in-person appointments were scheduled during March 23 and April 13, 2020 but cancelled due to statutory arrangements to not to go outside home due to COVID-19	The study involved the initial decision of the participants to join a telepsychiatry program was launched by the Outpatient Psychiatry Clinics at the University of Michigan health care system	Both telephone and video visits were considered	Equity issues in terms of age, race, sex and type of insurance were analysed	The study revealed that patients aged ≥44 years were more likely than patients aged 0-44 years to opt for telephone visits compared to video visits (relative risk reduction [RRR] 1.2; 95% CI 1.06-1.35).	The study results summarize that patient age correlates with the choice of virtual visit type, with older adults more likely to choose telephone visits over video visits.	The findings of the study suggest that considering the challenges to patient-facing technologies can address health equity issues, particularly those related to age.

Shaw 2013 [34]	Reducing disparity in outcomes for immigrants with cancer: a qualitative assessment of the feasibility and acceptability of a culturally targeted telephone-based supportive care intervention	Australia	Patients and family members attending community-based Chinese-speaking or hospital-based Arabic-speaking cancer support groups in Sydney, Australia	Cross-sectional qualitative design	This qualitative research was conducted among the patients and family members attending community-based Chinese-speaking or hospital-based Arabic-speaking cancer support groups in Sydney, Australia.	The intervention comprises five telephone calls in the patients' native language commencing soon after diagnosis and then at 1, 2, 4 and 6 months after enrolment. The call scheduled in high unmet need, in adjuvant therapy and unplanned health service contact.	Telephone calls	Equity issues related to the Culturally and Linguistically Diverse (CALD) population.	The participants viewed the intervention favourably as a means of providing information and support in the patient's language. Cultural considerations included assurances of confidentiality, as cancer is not openly discussed within communities. An initial face-to-face contact was highlighted as the most important factor facilitating participation.	The study highlighted the importance to develop a culture sensitive telephone-based supportive care intervention for Arabic- and Chinese-speaking cancer patients.	Developing culture sensitive virtual care intervention would be useful to ensure access of CLAD population in virtual care interventions. It can also be effective to initiate the first contact face-to-face.

Spooner 2017 [50]	eHealth patient-provider communication in the United States: interest, inequalities, and predictors	USA	This study used data from the Health Information National Trends Survey (HINTS) 2014, conducted by the National Cancer Institute	Cross-sectional	The participants for the study were adults population aged 18 years or more and present analysis was carried out among 3677 participants (HINTS 4 Cycle 4 data)	N/A	Interest to virtual care services include online patient-provider communication	Equity issues were analysed in terms of age, race, education, internet use and socio-economic status	The study revealed that younger respondents (<50 years), Hispanics, and those from higher income households were more likely to be interested in online PPC. It was also notable that, Internet users (odds ratio, OR= 2.87, 95% CI, 1.35-6.08), and college graduates (OR= 2.92, 95% CI, 1.42-5.99) had a higher likelihood of online PPC via email or fax, while Hispanics and those from higher-income households were 2–3 times more likely to communicate via text messaging or phone/mobile apps.	The study summarizes that patients' online communication with providers can be varied by age, race/ethnicity, education, income, and Internet access/behaviours.	This study once again poses the importance of increasing e-health literacy and special effort need to be given on older adults, ethnic minorities and socio-economically deprived population group.
Tam 2020 [51]	Disparities in the Uptake of Telemedicine During the COVID-	USA	The study was conducted in Henry Ford Health System	Cross-sectional	A total of 401 patient encounters were analysed and the patients were aged	Visit types included (1) virtual visits (completed using live audio and video), (2) telephone	Virtual visit and telephone visit	Equity issues considered was age, sex, household income and	In the multivariate analysis Medicaid/none/other public insurances (odds ratio [OR], 0.26; 95% CI], 0.10-0.66) and low median household income (second quartile OR, 0.33; 95% CI, 0.14-0.82; lowest quartile OR, 0.22;	This study particularly pointed the importance of insurance status and family income as a	While ensuring equity in virtual care intervention it needs to be considered

	19 Surge in a Multidisciplinary Head and Neck Cancer Population by Patient Demographic Characteristics and Socioeconomic Status		(Detroit, Michigan)		18 years and older with a head and neck cancer-related diagnosis evaluated between March 17 to April 24, 2020.	visits (completed only using telephone), (3) in-person visits, and (4) no-show visits.		insurance status	95% CI, 0.07-0.74) had lower completion of virtual visits.	determinant of virtual visit	that strategies are being developed to increase access of socio-economically vulnerable population.
Tong 2020 [52]	Identifying the barriers and perceptions of non-Hispanic black and Hispanic/	USA	This study was conducted among the patients from an ongoing randomized controlled	Cross-sectional qualitative design	This study involves conducting of eight semi-structured interviews comprised of open-ended	N/A	N/A	This study highlighted racial inequity in terms of access to virtual care	The study identified several factors that was responsible for not accessing the virtual care service such as disinterest (47%), inconvenience (33%), lack of perceived benefit (13%), lack of awareness of diabetes diagnosis (7%) and perceived lack of ability to	This study summarizes some of the important factors that are responsible for limited access to virtual care services such as disinterest,	To ensure racial equity in virtual care intervention greater awareness and understanding of these

	Latino persons with uncontrolled type 2 diabetes for participation in a home Telemonitoring feasibility study: a quantitative analysis of those who declined participation, withdrew or were non-adherent		trial (RCT) being conducted at Northwell Health, 'Feasibility of virtual care Management of Diabetes Mellitus type 2 (T2DM) in Black and Hispanic Minority Patients'.		questions and prompts conducted by telephone. The participants were patients purposively selected from an ongoing randomized controlled trial (RCT) being conducted at Northwell Health.			intervention	fully participate in the study (7%).	inconvenience and lack of perceived benefit.	issues will be critical.

Trief 2013 [67]	Adherence to diabetes self-care for white, African-American and Hispanic American telemedicine participants: 5 year results from the IDEATel project	USA	The study was carried out in SUNY Upstate Medical University, Syracuse, New York and Columbia University in New York City (NYC)	RCT	Participants (n=1665) recruited through primary care providers (PCPs) and included if receiving Medicare benefits, were 55 years of age, and diagnosed with diabetes. 821 randomized to usual care, and 844 to the telemedicine intervention.	The Informatics for Diabetes Education and Telemedicine (IDEATel), evaluated feasibility, acceptability, and effectiveness of a home telemedicine intervention in ethnically diverse, medically underserved, older adults with diabetes.	The intervention involved regular (every 46 weeks throughout the 5 years of the project) tele-visits with nurse case managers and dietitians via a home telemedicine unit (HTU), consisted of a web-enabled computer with a camera for the video visits.	Racial inequity was analysed in terms of adherence to the virtual care intervention.	Hispanic and African-American participants (p<0.001 for both) spent fewer days performing diabetes self-care activities overall (controlling for all covariates). Also, participants who had with more education (p=0.002) spent more days performing diabetes self-care activities	The findings summarized that African-American and Hispanic American participants were less adherent than white participants at all time points despite an individualized and accessible intervention. Also, lesser education exacerbated the condition.	It is important to consider the racial differences in adherence to delivered virtual care intervention. It is also important to consider how adherence can be improved among less educated participants.

van Veen 2019 [53]	Potential of Mobile Health Technology to Reduce Health Disparities in Underserved Communities	USA	This study was carried out in an urban emergency department (ED) in Detroit, Michigan	Cross-sectional	A total of 560 patients participated in the study. Most of the patients were adults (449, 80%) and 360 (64%) were female.	N/A	N/A	Equity issues considered based on sex and digital device.	The study found that adults were less likely to have access to phone consultation than parents of children (odds ratio [OR] 0.49, 95% confidence interval [CI], 0.32 – 0.74), as were males compared to females (OR 0.52, 95% CI, 0.37– 0.74). Most participants (92%) indicated that they would use a mHealth application.	The study highlighted that there is huge opportunity to scale up virtual care intervention. However, the study identified that phone consultations can be limited to males and adult patients.	More emphasis should be given to engage more male and adult patients in virtual care intervention if that is the case with virtual care interventions.
Walker 2020 [68]	Exploring the Digital Divide: Age and Race Disparities in Use of an Inpatient Portal	USA	This study was conducted at a large Midwestern academic medical center (AMC) that provides services across the	RCT	The study was conducted among 842 patients selected from six hospitals affiliated with a large, Midwestern	The patients were provided with access to a tablet equipped with an inpatient portal and recruited to participate in the study.	In-patients portal	Equity issues in terms of age and race was explored	The study revealed disparities in use of the inpatient portal in terms of age and race. Patients aged 60–69 (45.3% difference, $p < 0.001$) and those over age 70 (36.7% difference, $p = 0.04$) used the inpatient portal less than patients aged 18–29. Moreover, African American patients used the portal less than	The findings of the present suggest that access to technology may not be the only barrier that needs to be addressed to reduce the digital divide in terms of using patient portal	This study findings share a crucial message that only making access to the technology may not ensure equity in

			continuum of care at six hospitals.		academic medical center from July 2017 to July 2018.				White patients (40.4% difference, p = 0.004).	use. It is also important to address other barriers to reduce the digital divide.	service delivery. Promoting culture sensitive intervention, educational component to increase virtual care literacy and support older people through providing the assistance of educated caregiver can be of higher importance.
Wang 2018 [54]	Immigrants' Use of eHealth Services in the United States,	USA	This study used data from the adult sample of the 2011-2015	Cross-sectional	A total of 156355 adult respondents aged 18 years and above from	The outcomes of the study included 3 self-reported uses of eHealth	Telephone and online virtual care services	Equity issues based on immigration status was analysed.	The study findings revealed that, 18763 US natives (16.1%) reported using any eHealth services in the past 12 months, compared with 1738 (13.0%) naturalized citizens and 1020 (7.8%)	The study concludes that inequity exists in terms of using eHealth services among the immigrants that	This study pointed targeted intervention in virtual care specs to address

	National Health Interview Survey, 2011-2015		National Health Interview Survey (NHIS).		the 2011-2015 National Health Interview Survey (NHIS) took part in the study.	services: making medical appointments online, refilling prescriptions online, and communicating with health care providers through email.			noncitizens. Adjusting for socioeconomic factors reduced initial gaps: naturalized citizens (adjusted odds ratio [aOR] = 0.81; 95% confidence interval [CI], 0.75-0.87) and noncitizens (aOR = 0.81; 95% CI, 0.72-0.90) had approximately 20% lower odds of using eHealth services than did US natives. Als, Immigrants with higher English-language proficiency were more likely to use eHealth services than were immigrants with lower English-language proficiency.	would require targeted intervention address.	Inequity in terms of immigration status. Multilingual and culture sensitive portal use can be of value in this regard.
Weber 2020 [55]	Characteristics of virtual care users in NYC for COVID-related care	USA	This study used data from a large health system in NYC, the Mount Sinai	Cross-sectional	This study analysed 76 845 encounters for 52 585 unique patients diagnosed with, tested	The study analysed virtual care encounters versus ER encounters versus office visits	Not clearly explained	Equity issues pertaining to demographic factors such as race/ethn	The study findings revealed that, compared to Whites, Blacks had higher adjusted odds of using both the ER versus virtual care (OR: 4.3, 95% CI: 4.0-4.6) and office visits versus virtual care (OR: 1.4, 95% CI: 1.3-1.5). For	The study highlighted racial and age-related disparities in access to virtual care services compared to in-person services	Older adults and CALD people are subjected to the digital divide. It is therefore recommended to look for

n	during the coronavirus pandemic		deidentified COVID-19 database The data contain all patients diagnosed with, placed under investigation for, or screened negative for COVID-19 with any Mount Sinai system provider starting March 20, 2020.		for, or placed under investigation for COVID 19 between March 20 and May 18, 2020.			icity and age were analysed	Hispanics versus Whites, the analogous ORs were 2.5 (95% CI: 2.3-2.7) and 1.2 (95% CI: 1.1-1.3). Compared to any age groups, patients 65+ had significantly higher odds of using either ER or office visits versus virtual care.	amid this COVID-19 pandemic	strategies to address this digital divide.
Wegermann	Black, older, unmarried, and	USA	This study was performed in the	Retrospective cohor	A total of 13,628 visit attempts by adult	All patients were offered video visits first, and if	Both video visit and telephone	Equity issues related to race and	The study revealed that Black race/ethnicity was associated with increased odds of completion of a	The study summarized that vulnerable populations	Widespread disparities can exist among the

2020 [66]	medicaid patients were less likely to complete hepatolo gy video visits during COVID-19		hepatology clinics at Duke University Health System from January 1, 2020, through May 30, 2020	t study.	patients were analysed for the study. Of these, 3238 took place during the pre-COVID period, 3771 during the COVID period, and 6619 were outside study timeframe.	the patient was unable to complete a video visit or declined, a telephone visit was offered.	visit were considered	socioeco nomic character istics were analysed	telephone over a video visit, compared to White (OR=1.99, 95% CI 1.47, 2.68). It was also found that increasing age was associated with higher odds of a phone or incomplete visit (cancelled, no-show, or rescheduled after May 30,2020). It was also found that being single or previously married (separated, divorced, widowed) was associated with increased odds of completing a phone compared to video visit compared to being married.	including those that are older, non-Hispanic Black had lesser use or suboptimal use (phone versus video) of virtual care interventions during this COVID-19.	vulnerable populations particularly among aged and ethnic minorities that need to be addressed to ensure equity in virtual care intervention.
